# Comparative Prognostic Value of the National Institutes of Health Stroke Scale (NIHSS) and the Glasgow Coma Scale (GCS) in Supratentorial and Infratentorial Stroke Patients in Western India

**DOI:** 10.7759/cureus.65778

**Published:** 2024-07-30

**Authors:** Vishal Padwale, Chidanand Chivate, Vijendra Kirnake, Harshal Patil, Sunil Kumar, Nikhil Pantbalekundri

**Affiliations:** 1 Department of Medical Gastroenterology, Jawaharlal Nehru Medical College, Datta Meghe Institute of Higher Education & Research, Wardha, IND; 2 Department of Internal Medicine, Bharati Vidyapeeth (Deemed to be University) Medical College & Hospital, Sangli, Sangli, IND; 3 Department of Medicine, Jawaharlal Nehru Medical College, Datta Meghe Institute of Higher Education & Research, Wardha, IND

**Keywords:** mortality, morbidity, prognosis, gcs, nihss, infratentorial, supratentorial, stroke

## Abstract

Background

Acute coronary syndrome is the most common cause of mortality; cerebral vascular accident ranks second. Stroke is the fourth most common cause of disability worldwide, with nearly 20 million people suffering a stroke every year around the world and an estimated five million dead. Slightly more than 85.5% of stroke-related deaths take place in developing countries. In short, blockage (thrombus or emboli) and decreased blood supply for cerebral tissues lead to a stroke that permanently damages brain tissue. A stroke is clinically defined as rapidly developing clinical symptoms of focal cerebral dysfunction lasting >24 hours or leading to death, as characterized by the World Health Organization (WHO).

Objective

The present study was designed to compare the efficacy of the National Institutes of Health Stroke Scale (NIHSS) and the Glasgow Coma Scale (GCS) in determining the prognosis of supratentorial and infratentorial stroke.

Methods

This observational prospective study was performed on over 100 patients admitted to Bharati Hospital, Sangli, who had cerebrovascular accidents from February 2018 to June 2019. Eligibility criteria were adults more than 18 years of age with clinical and computed tomography/magnet resonance imaging (CT/MRI) evidence consistent with acute stroke. Trauma and concomitant supra- and infratentorial strokes were excluded. GCS and NIH stroke scale scores were measured daily, and scores were noted on the first and last day of hospitalization. Statistical analysis was done using IBM SPSS Statistics for Windows, Version 22 (Released 2013; IBM Corp., Armonk, New York, United States), including mean, standard deviation, paired t-test, and Chi-square test.

Results

Out of 100 patients, 77% had suffered supratentorial strokes, and thus the other 23% had infratentorial strokes. Alcohol consumption was associated with a higher risk of infratentorial strokes, while smoking was linked to a higher risk of supratentorial strokes. Diabetes and hypertension did not differ statistically between the two groups. Compared to patients with supratentorial strokes, those who suffered from infratentorial strokes had a greater death rate and less favorable recovery results. Patients with supratentorial strokes who recovered completely or partially showed considerable improvements in their GCS scores, but patients with infratentorial strokes showed minimal to no improvement. On the other hand, the NIHSS score significantly improved in patients who achieved both complete or partial recovery and no improvement or mortality in both supratentorial and infratentorial stroke. NIHSS is preferred over GCS because it provides a better insight into morbidity and neurological outcomes of both types of strokes in comparison with GCS, which is more useful in predicting mortalities.

Conclusion

According to this study, supratentorial strokes were more common, whereas infratentorial strokes had a worse prognosis. Alcohol ingestion and smoking may have an impact on the location of a stroke. Compared to GCS, the NIHSS score provided a more thorough evaluation of stroke recovery, indicating its potential for better patient care.

## Introduction

Acute coronary syndrome is the most common cause of mortality; cerebral vascular accident ranks second. It ranks as the fourth most prevalent primary cause of disability globally. Every year, stroke affects around 20 million people, and five million of them pass away. Although the number of stroke-related deaths is declining in the industrialized world, 85.5% of all stroke deaths happen in developing nations. Stroke morbidity was around seven times higher in underdeveloped than developed nations [1].

Thrombus or emboli cause a decreased or absent vascular supply to the cerebral tissues, which is what causes an ischemic stroke. The oxygen and glucose are cut off by this reduction in blood flow, permanently harming the brain parenchyma's tissues. "Rapidly developing clinical symptoms and signs of focal cerebral disturbance lasting more than 24 hours or leading to death with no apparent cause other than vascular origin" is how the World Health Organization (WHO) defines stroke clinically [2,3].

A typical ischemic stroke is clinically characterized by its mode of onset and subsequent course. It often manifests as an abrupt start of a localized neurological impairment. The signs and symptoms, as well as indications of interventions depending on an ischemic stroke, are dependent on the position of the blood artery blockage and the extent of peripheral flow. The hallmark of stroke is sudden onset hemiparesis in an individual [4-6].

These days, stroke syndromes are further divided into supratentorial and infratentorial strokes according to the area of the brain parenchyma affected and where it is in relation to the tentorium cerebelli. Supratentorial compartment stroke comprises the stroke due to occlusion of the internal carotid artery and its branches. Conversely, infratentorial compartment syndrome refers to a stroke brought on by the vertebral artery and its branch obstruction. Infratentorial stroke accounts for approximately 20% of all strokes, and it is due to posterior circulation thrombosis or embolism [7-9].

Determining the disease's prognosis requires a precise assessment of the disease's severity. Acute Physiology and Chronic Health Evaluation (APACHE) II and III, the Glasgow Coma Scale (GCS) score, the Chinese Stroke Scale (CSS), the National Institutes of Health Stroke Score (NIHSS), and the activities of daily living (ADL) (Barthel Index, BI) are among the various tests that are employed. The grading systems are used to assess the degree of illness and forecast the likelihood of disease.

The most popular rating method for determining a stroke's severity is the NIHSS. It is primarily used to assess prognosis, therapeutic impact, and the degree of neurological damage. The GCS scale, often known as the coma scale, was created in 1978 by Teasdale et al. for patients undergoing brain surgery. It is typically used to examine the functional abnormalities of the neurological system and the degree of consciousness of stroke patients [10,11].

By providing clinicians the greatest possible assistance in diagnosing and treating stroke patients, comparison of stroke scales improves the overall quality of stroke care. It will also highlight each one's weaknesses and advantages, pointing out areas in need of development or further study [12]. Very few studies compare NIHSS and GCS scoring systems to determine the prognosis of supratentorial and infratentorial strokes. Thus, the present study compared NIHSS and GCS systems to determine the prognosis, morbidity, and mortality of supratentorial and infratentorial strokes.

## Materials and methods

This observational prospective study included a total of 100 individuals with cerebrovascular accidents who were admitted to Bharati Hospital, Sangli, India, and placed in the medicine ward or intensive care unit (ICU) and who met the study's inclusion criteria. The research was carried out between February 2018 and June 2019. Informed written consent was obtained from patients and their relatives in their language. The study included patients older than 18 years of age with strong clinical evidence of cerebrovascular accident, as well as those with computed tomography (CT) or magnetic resonance imaging (MRI) evidence of cerebrovascular accident. Patients with extradural hemorrhages, subdural hemorrhages, subarachnoid hemorrhages, and cerebral hemorrhages due to any traumatic cause and patients with simultaneous occurrence of both supratentorial and infratentorial cerebrovascular accidents were excluded from the study.

A detailed history was taken from patients and, in some cases, from reliable relatives of the patients. Only while the patient was in the hospital were all cases followed. All laboratory tests, clinical characteristics, and examinations were finished and recorded on proformas. Every clinical symptom and indication was taken into account. In addition to noting CT or MRI results for every patient and entering them into proformas, GCS and NIHS scores have been calculated and recorded onto the proforma provided on the first day. Cases were followed daily with reference to the Glasgow Coma Scale, National Institute of Health Stroke Scale (NIHSS), CT and MRI findings, day-to-day improvement or deterioration, need for mechanical ventilation, and weaning. All patients underwent CT and/or MRI on the first day of admission and/or in between during the hospital stay. Final observations were made on the day of discharge, that is, the last day, concerning the outcome of patients and all clinical features in the form of scoring systems. All details about every case were entered in the master chart. Simple mean, median, mode, standard deviation, unpaired t-test, and Chi-square test were applied for tabulated data analysis. The chi-square value was calculated for a degree of freedom and a probability of 0.05. The null hypothesis was accepted if the computed value was less than the tabulated value.

## Results

Among these completely recovered cases, the GCS scores improved significantly from 11.65 ± 0.79 on the first day to 14.24 ± 0.44 on the last day (p < 0.001) in supratentorial stroke. The GCS scores increased from 11.75 ± 0.50 on the first day to 12.75 ± 0.50 on the last day, but this change was not statistically significant (p = 0.124) in infratentorial strokes. Among partially recovered cases, there was a significant improvement in GCS scores from 9.31 ± 1.13 on the first day to 11.83 ± 1.22 on the last day (p < 0.001) in supratentorial strokes. The GCS scores increased slightly from 11.20 ± 1.10 on the first day to 11.30 ± 1.10 on the last day, but the change was not statistically significant (p = 0.345) in infratentorial strokes. Among cases with no improvement, the GCS scores decreased from 7.89 ± 0.76 on the first day to 7 ± 1.28 on the last day, indicating a significant decline (p = 0.025). The GCS scores significantly declined from 10 ± 0 on the first day to 5 ± 0 on the last day (p = 0.05) in infratentorial stroke. Among the cases with mortality, the GCS scores decreased from 8.29 ± 0.76 on the first day to 6.14 ± 2.97 on the last day, which is marginally significant (p = 0.05) in supratentorial stroke. The GCS scores significantly declined from 9.15 ± 1.28 on the first day to 3.69 ± 0.75 on the last day (p < 0.001) in infratentorial stroke (Table 1).

**Table 1:** Utility of mean of GCS score within outcome with diagnosis (n=100); SD: standard deviation: GCS: Glasgow Coma Scale

GCS	Supratentorial strokes (N=77)		Infratentorial strokes (N=23)	
	Mean ±SD	P-value (Paired t-test)	Mean ±SD	P-value (Paired t-test)
Completely recovered				
1^st^ day	11.65± 0.79	(Baseline)	11.75± 0.50	(Baseline)
Last day	14.24 ± 0.44	<0.001	12.75 ± 0.50	0.124
Partially recovered				
1^st^ day	9.31 ± 1.13	(Baseline)	11.20 ± 1.10	(Baseline)
Last day	11.83 ± 1.22	<0.001	11.30 ± 1.10	0.345
No improvement				
1^st^ day	7.89 ± 0.76	(Baseline)	10 ± 0	(Baseline)
Last day	7 ± 1.28	0.025	5 ± 0	0.05
Death				
1^st^ day	8.29 ± 0.76	(Baseline)	9.15 ± 1.28	(Baseline)
Last day	6.14 ± 2.97	0.05	3.69 ± 0.75	<0.001

Among those completely recovered, the NIHSS scores improved significantly from 12.41 ± 1.46 on the first day to 7.88 ± 1.11 on the last day (p < 0.001) in supratentorial strokes. The NIHSS scores significantly improved from 12 ± 1.63 on the first day to 7.50 ± 0.58 on the last day (p = 0.014) in infratentorial strokes. Among partially recovered, there was a significant improvement in NIHSS scores from 15.29 ± 1.90 on the first day to 11.23 ± 1.93 on the last day (p < 0.001) in supratentorial strokes. The NIHSS scores significantly improved from 14 ± 2 on the first day to 9 ± 1.23 on the last day (p = 0.004) in infratentorial strokes. In cases with no improvement, the NIHSS scores increased from 16 ± 1.24 on the first day to 19 ± 3.05 on the last day, indicating a significant deterioration (p = 0.002) in supratentorial strokes, while the NIHSS scores significantly deteriorated from 12 ± 0 on the first day to 18 ± 0 on the last day (p < 0.001) in infratentorial strokes. In cases with mortality, the NIHSS scores increased from 14.71 ± 1.89 on the first day to 19.29 ± 4.42 on the last day, indicating a significant deterioration (p = 0.035) in supratentorial strokes, while the NIHSS scores significantly worsened from 12 ± 2.12 on the first day to 21.85 ± 2.85 on the last day (p < 0.001) in infratentorial strokes (Table 2).

**Table 2:** Utility of mean of NIHSS score within outcome with diagnosis (n=100); SD: standard deviation; NIHSS: National Institutes of Health Stroke Scale

NIHSS score	Supratentorial strokes (N=77)		Infratentorial strokes (N=23)	
	Mean ±SD	P value (Paired t-test)	Mean ±SD	P value (Paired t-test)
Completely recovered				
1^st^ day	12.41± 1.46	(Baseline)	12± 1.63	(Baseline)
Last day	7.88 ± 1.11	<0.001	7.50 ± 0.58	0.014
Partially recovered				
1^st^ day	15.29 ± 1.90	(Baseline)	14 ± 2	(Baseline)
Last day	11.23 ± 1.93	<0.001	9 ± 1.23	0.004
No improvement				
1^st^ day	16 ± 1.24	(Baseline)	12 ± 0	(Baseline)
Last day	19 ± 3.05	0.002	18 ± 0	<0.001
Death				
1^st^ day	14.71 ± 1.89	(Baseline)	12 ± 2.12	(Baseline)
Last day	19.29 ± 4.42	0.035	21.85 ± 2.85	<0.001

## Discussion

Severity ratings are essential therapeutic adjuncts in the ICU for projecting patient outcomes, evaluating healthcare quality, and stratifying clinical research. They are crucial for improving medical decision-making and detecting patients with unexpected results. The prediction models can be applied successfully despite some difficulties. The GCS grading system has received approval for benchmarking and mortality prediction in neurological patients. Healthcare practitioners utilize the National Institutes of Health Stroke Scale (NIHSS) to evaluate the damage caused by a stroke objectively. The NIHSS consists of 11 components rated for different abilities on a scale ranging from zero to four. Each item has a score; a higher number implies some impairment, whereas a score of zero indicates a normal function in that particular ability [13].

In the present study, a total of 100 patients with sudden onset acute ischemic stroke were included in the study. Out of 100 patients, 77% were supratentorial stroke cases, and 23% were infratentorial stroke cases. Thus, in the present study, supratentorial stroke cases were three times more common than the infratentorial cases. These results are based on the survey by Libman et al. [14]. In his study, 86% were supratentorial stroke cases, and 14% were infratentorial stroke cases, where supratentorial cases were three times more common than infratentorial cases. In the present study, 68.8% of supratentorial stroke patients and 82.6% of infratentorial stroke patients had hypertension, which is found to be the single most important risk factor associated with stroke. This result nearly correlates with the Banerjee et al. [15] study conducted in Calcutta on an urban population where systemic hypertension emerged as the single most important risk factor in 62%. The study emphasizes the strong correlation between smoking, alcohol use, and various forms of stroke. Alcohol consumption is more common among patients with infratentorial strokes, whereas smoking is more prevalent among those with supratentorial strokes. The prevalence of diabetes mellitus and hypertension, however, did not differ significantly between the two types of strokes. These results imply that smoking and alcohol use may be important lifestyle factors that influence the kind of stroke a patient is most likely to suffer.

In contrast to infratentorial strokes, supratentorial strokes had superior recovery rates (both complete and partial), whereas infratentorial strokes presented a markedly elevated mortality rate. Both GCS and NIHSS show significant improvements, indicating these scales are reliable in detecting recovery in stroke patients. Perhaps the GCS system was not consistent for infratentorial strokes in detecting complete recovery and no recovery, suggesting that the location of the stroke might influence the sensitivity of these scales. Like infratentorial stroke, cases have a significant amount of cerebellar infarction whose further prognosis is uncertain. Both scales effectively capture mortality, with NIHSS providing a more detailed assessment of neurological deterioration [16]. We found that GCS precisely described outcomes in both supratentorial and infratentorial strokes as a mortality indicator. It did not, however, identify worse neurological outcomes on the first and last day in nearly all cases of infratentorial stroke and very few cases of supratentorial stroke. To identify morbidity or mortality markers, we compared the NIHSS scores of patients who had supratentorial and infratentorial strokes for their first and last day's outcomes. In this instance, we discovered that in both supratentorial and infratentorial stroke cases, the NIHSS score significantly outperformed the GCS score in identifying worse neurological outcomes on the first and last day [17,18]. The NIHSS score simultaneously identifies mortality indicators in supratentorial and infratentorial stroke, such as the GCS score [19].

However, this study has a few limitations. Firstly, it was conducted in one of western India's tertiary care centers. Secondly, the sample population was small, which limits the generalizability of the findings. Also, the study did not attempt to differentiate large vessel or cortical stroke from small vessel or lacunar type stroke, which might impact analysis and outcomes in relation to the GCS and NIHSS scores.

## Conclusions

The present study shows that the incidence of supratentorial stroke is higher than infratentorial stroke. These results imply that smoking and alcohol use may be important lifestyle factors that influence the kind of stroke a patient is most likely to suffer. Infratentorial stroke has poorer outcomes than supratentorial strokes, but the morbidity of supratentorial stroke is higher than infratentorial strokes. NIHSS is generally more sensitive and comprehensive in assessing stroke severity and recovery than GCS. However, it has limitations because it is less sensitive in infratentorial stroke. It can capture significant changes in both supratentorial and infratentorial strokes, whereas, in comparison, GCS may miss some improvements, especially in infratentorial strokes. GCS score and NIHSS score are important in predicting mortality and morbidity. Thereby, patient counseling becomes an easy job. The utility of the NIHSS score is higher in defining the outcomes of both supratentorial and infratentorial stroke in the form of morbidity, mortality, and poor neurological outcomes.

In contrast, the GCS score is useful only for defining poorer outcomes and mortality outcomes. In infratentorial stroke, an anatomical region with a greater propensity for alterations in levels of consciousness might certainly be expected to be lower, and depending on the type of stroke (large vessel or small vessel), the recovery might vary in GCS score. Therefore, despite its limitations, NIHSS might be preferred for a detailed neurological assessment in stroke patients compared with the GCS scoring system.
